# Chronic Sleep Disruption Advances the Temporal Progression of Tauopathy in P301S Mutant Mice

**DOI:** 10.1523/JNEUROSCI.0275-18.2018

**Published:** 2018-11-28

**Authors:** Yan Zhu, Guanxia Zhan, Polina Fenik, Madison Brandes, Patrick Bell, Noelle Francois, Katherine Shulman, Sigrid Veasey

**Affiliations:** Center for Sleep and Circadian Neurobiology and Department of Medicine, Perelman School of Medicine, University of Pennsylvania, Philadelphia, Pennsylvania 19104

**Keywords:** Gallayas, locus ceruleus, sleep deprivation, tauopathy

## Abstract

Brainstem locus ceruleus neurons (LCn) are among the first neurons across the lifespan to evidence tau pathology, and LCn are implicated in tau propagation throughout the cortices. Yet, events influencing LCn tau are poorly understood. Activated persistently across wakefulness, LCn experience significant metabolic stress in response to chronic short sleep (CSS). Here we explored whether CSS influences LCn tau and the biochemical, neuroanatomical, and/or behavioral progression of tauopathy in male and female P301S mice. CSS in early adult life advanced the temporal progression of neurobehavioral impairments and resulted in a lasting increase in soluble tau oligomers. Intriguingly, CSS resulted in an early increase in AT8 and MC1 tau pathology in the LC. Over time tau pathology, including tangles, was evident in forebrain tau-vulnerable regions. Sustained microglial and astrocytic activation was observed as well. Remarkably, CSS resulted in significant loss of neurons in the two regions examined: the basolateral amygdala and LC. A second, distinct form of chronic sleep disruption, fragmentation of sleep, during early adult life also increased tau deposition and imparted early neurobehavioral impairment. Collectively, the findings demonstrate that early life sleep disruption has important lasting effects on the temporal progression in P301S mice, influencing tau pathology and hastening neurodegeneration, neuroinflammation, and neurobehavioral impairments.

**SIGNIFICANCE STATEMENT** Chronic short sleep (CSS) is pervasive in modern society. Here, we found that early life CSS influences behavioral, biochemical, and neuroanatomic aspects of the temporal progression of tauopathy in a mouse model of the P301S tau mutation. Specifically, CSS hastened the onset of motor impairment and resulted in a greater loss of neurons in both the locus ceruleus and basolateral/lateral amygdala. Importantly, despite a protracted recovery opportunity after CSS, mice evidenced a sustained increase in pathogenic tau oligomers, and increased pathogenic tau in the locus ceruleus and limbic system nuclei. These findings unveil early life sleep habits as an important determinant in the progression of tauopathy.

## Introduction

Brainstem locus ceruleus neurons (LCn) are sole providers of noradrenaline to the cortices (Foote et al., 1983), and these neurons help coordinate increased neuronal activity with regional blood flow and glial responses that are critical to optimal cognitive performance and brain health (Aston-Jones et al., 1994; Usher et al., 1999; Strange and Dolan, 2004; Sara, 2009; Bekar et al., 2012). LCn, however, evidence heightened susceptibility in many neurodegenerative processes (Braak et al., 2011). Degeneration of LCn is evident in mild cognitive impairment and early Alzheimer's disease (AD), and the extent of LCn loss predicts cognitive decline (Kelly et al., 2017). LCn seem particularly vulnerable in several tauopathies, including AD. Indeed, somatodendritic tau tangles may be observed in LCn decades before deposition of cortical tangles or clinical AD (Braak et al., 2011). Interestingly, injection of pathogenic tau preformed fibrils into the hippocampus (HC) and other forebrain regions leads to a pronounced concentration of tau aggregates in LCn, relative to other brainstem ascending neuronal groups (Iba et al., 2013), and injection of preformed fibrils into the LC results in widespread cortical tau pathology (Iba et al., 2015). In addition to LC vulnerability in tauopathies, injury to the LC can accelerate the temporal progression of pathology in some murine models of AD and Down's syndrome (Lockrow et al., 2011), whereas lesioning LCn in the P301S murine model of tauopathy hastens cognitive decline and gliosis (Chalermpalanupap et al., 2018). Collectively, these findings suggest important feedforward influences between LCn injury and the progression of tauopathy.

Chronic sleep disruption can impart LCn injury and, thus, could influence the progression of tauopathies. Both chronic short sleep (CSS) and chronic fragmentation of sleep (CFS) in young adult mice induce lasting metabolic stress to and degeneration of LCn (Zhang et al., 2014; Zhu et al., 2015, 2016). Intriguingly, neuronal activation increases brain tau, tau pathology, and even propagation of tau via exosomes (Yamada et al., 2014; Wu et al., 2016; Wang et al., 2017). LCn demonstrate sustained heightened activity across wakefulness (Aston-Jones and Bloom, 1981), which could, therefore, influence LC tau. Importantly, while increased neuronal activity results in a rapid increase in tau (within hours), tau clearance from the interstitial space is protracted (Yamada et al., 2014). We therefore hypothesized that repeated daily exposures to CSS would result in an accumulation of LC tau and potentially induce important pathogenic post-translational modifications of tau, which in a murine model of tauopathy would increase tau pathology and hasten the temporal progression of tauopathy.

While LCn show early involvement in tauopathies, cortical tau likely contributes significantly to both forebrain neurodegeneration and neurobehavioral impairments. We therefore also examined the effects of CSS on tau in tauopathy-vulnerable rostral brain regions and determined whether CFS, as a distinct second form of sleep disruption, influences tau pathology and the temporal progression of behavioral impairment in the murine tauopathy model.

## Materials and Methods

### 

#### 

##### Animals.

Studies were performed at the University of Pennsylvania in accordance with the National Institutes of Health Office of Laboratory Animal Welfare Policy and the Institutional Animal Care and Use Committee at the University of Pennsylvania. Male and female mice expressing the human P301S tau mutation (PS19 strain) under regulation of the mouse prion promoter on a mixed C57BL/6NJ (B6) background (B6N.Cg-TgPrnP-MAPT^P301S^PS19Vle/J) were studied, along with WT littermates from hemizygous males and WT females bred in our colony. For each long-term recovery experiment numbers of males and females were equal across sleep conditions. A separate group of no recovery and age-matched rested controls was added, comprised of all males. Tissue from microtubule-associated protein tau knock-out B6.129X1-Mapt^tm1Hnd^/J (Tau^−/−^) mice served to substantiate specificities of tau antibodies. Mice were housed in a light/dark environment with lights on from 7:00 A.M. to 7:00 P.M. and fed *ad libitum* standard rodent chow and water throughout experimentation.

##### Chronic sleep disruption.

**Figure 1.**
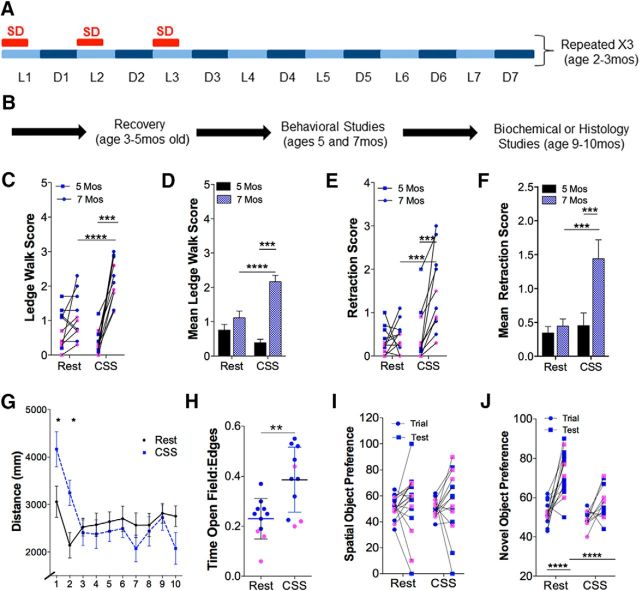
Temporal overview of study design and effects of CSS on neurobehavioral performance in P301S mutant mice. ***A***, Schematic of the CSS paradigm, where total sleep deprivation (SD, red bars) occurred at the onset of the first three lights-on (light blue) periods of the week (L1, L2, and L3) for 8 consecutive hours. Mice were returned to home cages for the last 4 h of the L1, L2, and L3 periods and the ensuing lights off (dark blue bars, D1, D2, and D3) periods. Mice were left undisturbed in home cages for days 4–7 each week. The pattern was repeated weekly for 4 consecutive weeks. ***B***, Following CSS and Rest control conditions, mice recovered 3 months before behavioral tests at ages 5 and 7 months and then an additional 2–3 months before undergoing the specified protein assays at ages 9–10 months. ***C***, ***D***, Individual and group (mean ± SE) scores for the ledge walk test, where higher scores, to a maximum of 3, indicate greater impairment (*n* = 11: 7 male, 4 female/group). Individual data points: blue represents male; pink represents female. ***E***, ***F***, Individual (same color scheme/gender) and group scores for hindlimb retraction, where scores to a maximum of 3 indicate impairment (*n* = 11: 7 male, 4 female/group). ***G***, Locomotor distance (mm) per minute in novel environment (mean ± SE) for 10 min in Rest (black) and CSS (blue) mice. *n* = 11 (8 male, 3 female/group). ***H***, Individual data points (blue represents male; pink represents female) for ratio of time spent in center of open field relative to edges (also *n* = 11: 8 male, 3 female/group). Error bars indicate mean ± SE. ***I***, Spatial object memory response expressed as percentage preference to moved object. *n* = 11 (8 male, 3 female/group). Individual data points, coded as above, with paired responses for before move (trial, circle) and after move (test, square). ***J***, Novel object memory test with paired individual data points, coded as above, for preference to original object (trial, circle) and novel object replacement (test, square). ***C–F***, ***I***, ***J***, Repeated-measures ANOVA. ***G***, Two-way ANOVA. ***H***, *t* test. *p < 0.05, **p < 0.01, ***p < 0.001, ****p < 0.0001.

Two distinct forms of sleep disruption were examined: CSS and CFS. CSS was achieved using a continuously monitored enriched environment in which novel climbing toys were periodically exchanged whenever a mouse became behaviorally quiescent (Vankov et al., 1995; Léger et al., 2009; Gompf et al., 2010). A temporal overview of the protocol and general experimental design is presented in Figure 1*A*. On days 1–3 of each of 4 consecutive weeks, mice were placed along with littermates in the novel environment with standard bedding, chow, and water bottles for the first 8 h of the lights-on period. Rested controls (Rest) were placed in a similar environment for the first hour of the lights-on period, a time when mice are generally awake. Ambient lighting and temperature were held similar to the home cage environment. Plasma corticosterone levels are not elevated in this chronic paradigm (Zhang et al., 2014). A second approach to chronic sleep disruption, CFS, was implemented to help distinguish tau and behavioral responses to disturbed sleep from responses secondary to an enriched environment and/or increased locomotor activity, present only in the CSS paradigm. Home mouse cages were placed atop a rotor table (rotation speed 1 Hz; 5 s every minute; 24 h/d for 4 weeks). This paradigm increases the arousal frequency from 30/h to 60/h without significantly influencing total wake or sleep time in 24 h (Zhu et al., 2015).

##### Neurobehavioral testing.

Motor and memory impairments, hyperactivity, and disinhibition have been identified early in P301S mice (Yoshiyama et al., 2007; Dumont et al., 2011; Takeuchi et al., 2011; Przybyla et al., 2016). The hindlimb retraction assay and ledge walk assessment tests were performed as previously described (Guyenet et al., 2010) on CSS and Rest mice 1 and 3 months after CSS (ages 5 and 7 months). Mice were habituated to handlers for 3 d before testing. Tests were scored on an integer scale 0–3, with 0 as normal performance and 3 as continuous clasping of hind limbs (limb retraction assay) or inability to move forward along the ledge (Guyenet et al., 2010). Each of these tests was repeated 4 times and an average score was used for the time point.

A second group of mice Rest and CSS P301S mice was assessed for open field activity, novel and spatial object recognition memory at 7 months (4 months after CSS or Rest conditions). Locomotor activity (distance traveled/min in open field) and percentage of time in center 10 cm × 10 cm were quantified, in an open field, measuring 50 × 50 cm^2^, and illuminated from above by 25 lux. Mice were placed individually into the arena and monitored for 10 min by a video camera (Sony CCD IRIS). Transitory data were analyzed with tracking using the image processing system EthoVision 3.1 (Noldus Information Technology). The novel and spatial object memory protocols were adapted from detailed published protocols (Sinha et al., 1999; Youmans et al., 2011). Mice were habituated to a 60 cm × 50 cm × 30 cm chamber without objects for 5 min for three consecutive mornings with lighting as above. The spatial object test was run 1 week and the novel object test the following week. For the spatial object test, on test day, mice received two 5 min training sessions, 30 min apart, followed by the test 3 h later in which one of two objects was moved. Between training and testing sessions, mice were left unperturbed in home cages. The only difference in the novel object test was that, during the test phase, one object was randomly replaced with a novel object. Scorers reviewing videos were blinded to the conditions and genotypes of mice. The percentage of time spent attending the original object relative to time spent attending the moved or novel object was determined for each mouse for both trial and test conditions.

##### Antibodies.

Primary antibodies used for characterization of immunoblot and immunohistochemistry responses were as follows: AT8 Tau (PSer202, Thr 205; MN1020, Thermo Fisher Scientific); P202 Tau (Pser202; 11834, Cell Signaling Technology); AT180 Tau (PThr231; MN1040, Thermo Fisher Scientific); MC1 Tau (kind gift from Peter Davies); TOMA-1 (oligomeric tau, MABN819, Millipore), Tau-5 (total tau; Ab80579, Abcam); CD68 (Ab125212, Abcam); ionized calcium binding adapter protein 1, Iba-1 (Ab107159, Abcam); GFAP (Ab7260, Abcam); and TH (LS-C124752, LSBio, and 22941, Immunostar).

##### Immunoblotting.

Mice designated for protein lysate assays were decapitated, and brains were frozen on dry ice, sectioned coronally 0.3- to 0.5-mm-thick on a cooling block, and then, using an #11 gauge scalpel and dissecting scope, LC and EC tissue was immediately excised and homogenized on ice in TBS lysis buffer with protease inhibitor mixture (P8340, Sigma), phosphatase inhibitor (Halt, 1862495, Thermo Fisher Scientific), and 1% Triton to improve capture of all tau (Sahara et al., 2002). LC-enriched samples were taken to include much of the LC nucleus and dendritic field but likely included some of the lateral dorsal tegmentum and mesencephalic trigeminal neurons, whereas EC-enriched samples may have included some of the external capsule. Protein extracts (20 μg/sample, BCA measured) were run on SDS-PAGE and transferred to nitrocellulose membranes. Loading buffer (TBS 927–50000, Odyssey) was used to enhance phosphorylated target protein signal. β-Mercaptoethanol and DTT were removed from loading buffer to capture oxidized tau oligomers (Kim et al., 2015). Gels were imaged and analyzed with Odyssey CLx Imager with Odyssey Application software, version 3.0.16 (Li-Cor). Mean integrated densities for 50–80 kDa and 90–160 kDa were normalized to α-tubulin, as total tau was altered by CSS.

##### Histochemistry and stereology.

Mice were anesthetized with pentobarbital and transcardially perfused with ice-cold PBS followed by 4% PFA. Brains were after fixed in PFA and cryopreserved before sectioning as 60-μm-thick serial coronal slices. Sections were selected (3/region/mouse) for LC (−5.20 to −5.80 bregma) (Franklin and Paxinos, 2008), BLA/lateral amygdala (−1.34 to −2.15 bregma), rostral HC (−1.22 to −2.54 bregma), and EC (−3.16 to −4.36 bregma). For light microscopy, free-floating 1% Triton-treated sections were processed as previously reported (Panossian et al., 2011) using the following titers of tau primary antibodies: AT8 (1:2500) or MC1 (1:500), TH (1:2000), or glial antibodies, Iba-1 (1:1000), CD68 (1:1000), and GFAP (1:500). Enzyme-linked immunohistochemistry was performed with biotinylated secondary antibodies and biotin-avidin peroxidase complex visualized with Vector Blue (Vector Laboratories). Imaging was performed with a DM5500B (Leica Microsystems). Using Fiji (ImageJ) software, images were converted to 8-bit grayscale and thresholded to intense labeling with user blinded to sleep conditions. Percentage area within target region was measured, and target area percentage coverage averages for each mouse were analyzed.

Unbiased optical fractionator stereology for neuronal count estimates, as previously detailed (West and Gundersen, 1990), was performed for LCn and BLA/LA nuclei. LCn were immunolabeled with anti-TH (LS-C124752). LCn and amygdala sections were mounted, dried, and counterstained with Giemsa (to identify neurons and nuclei), and sections were confirmed to span the entire rostral-caudal nucleus. A 100× oil objective was used to count neuronal nuclei in focus within the probe boundaries (DM4B, Leica Microsystems). For LCn, all neurons (TH^+^ and TH^−^) with soma > 10 μm diameter within the confines of the bilateral LC nuclei were counted using a probe size of 50 μm × 50 μm and a counting frame of 100 μm × 100 μm (StereoInvestigator version 11.09, MicroBrightField Biosystems). This strategy, using a 1:2 series of sections provided >200 counts, allowed a Gundersen coefficient of error (for m = 1) < 0.09 in all subjects. Anatomical boundaries of the amygdala subnuclei (lateral and basal amygdala) were delineated as previously described, using fiber tract landmarks and cytologic features of neurons within each group (Chareyron et al., 2011). A 1:3 series was examined, unilaterally, using a probe size of 35 μm × 35 μm and a counting frame of 185 μm × 185 μm. This allowed counts of 200–450 neurons per subject and a Gundersen <0.09 in all cases.

To identify argyrophilic pathology, including neurofibrillary tangles (NFTs) and neuropil threads, Gallyas silver impregnation was performed on mid LC and BLA/LA (−5.40 to −5.80 and −1.34 to −2.30 bregma, respectively) 60 μm mounted sections (2 or 3/region/mouse) as previously detailed (Kuninaka et al., 2015). Timing of developer exposure was optimized to provide absence of signal in WT mice and some strong neuronal labeling in 10-month-old male P301S mice in the amygdala and/or piriform cortices. Sections were imaged using the DM5500B microscope, before and after nuclear fast red counterstain (N8002, Sigma-Aldrich), and the percentage area of tangles was measured in the above regions, as above for AT8 and MC1 analyses using ImageJ.

Additional sections were analyzed for MC1 and GFAP responses in mice without prolonged recovery after CSS. Sections were processed as above, with the exception that secondary antibodies were conjugated with AlexaFluor probes: 488, 555, or 594 (Invitrogen) for visualization using confocal microscopy (SP5/AOBS, Leica Microsystems). Anti-TH labeling was used to highlight the LC region, and DAPI nuclear labeling was used to delineate hippocampal CA1. Confocal laser intensities, nanometer range, detector gain, exposure time, amplifier offset, and depth of the focal plane within sections per antigen target were standardized across compared sections (Panossian et al., 2011). Percentage area coverage in 2 sections of LC nucleus (−5.40 to −5.80 bregma) and the CA1 (−1.22 to −2.54 bregma) regions was assessed for MC1 and GFAP, using 8-bit grayscale inverted montaged images across 17 μm *z* axis, standardized thresholds with average percentage areas obtained per section/mouse and analyzed across groups.

##### Statistical analysis.

When a single variable was compared across two groups, the Student's *t* test (unpaired) was implemented with Bonferroni correction for multiple comparisons; and when three groups were compared, one-way ANOVA with Bonferroni's multiple comparisons test was used. Within-animal memory testing was performed using repeated-measures two-way ANOVA with Holm–Sidak multiple-comparison test. Repeated-measures ANOVA was also used to assess changes within animal in neurobehavioral performance over time, using Tukey's multiple-comparison analysis for overall significant interaction(s). For comparisons of >2 groups across genotype and sleep conditions, two-way ANOVA was used with Tukey's multiple-comparison *post hoc* analyses. The cutoff for significant statistical power for all analyses was a multiple-comparisons-corrected *p* < 0.05.

## Results

### Hastened neurobehavioral deterioration in P301S mice following CSS

We first determined whether early-life CSS would influence the progression of known neurobehavioral impairments in P301S mice by assessing motor behavior and spatial memory (Yoshiyama et al., 2007; Xu et al., 2014). Motor performance was examined at ages 5 and 7 months (1 and 3 months after CSS or Rest conditions), using a ledge walk test and the hindlimb retraction test, both analyzed as *n* = 11 (7 male, 4 female)/group. The former assesses agility of movement, and the latter is one of the earliest motor deficits observed in P301S mice (Yoshiyama et al., 2007). Overall, there were both age and sleep condition effects on ledge walking ability. Individual and group data are presented in Figure 1*C*, *D*. In Rest mice, ledge scores were unchanged from 5 to 7 months of age (*t* = 1.5, not significant); whereas in CSS mice, ledge scores deteriorated from 5 to 7 months (*t* = 4.5, *p* < 0.001). Although there was no effect of sleep condition on the ledge test performance at age 5 months (*t* = 1.6, not significant), CSS mice age at 7 months showed poorer performances, relative to age-matched Rest mice (*t* = 8.4, *p* < 0.0001). Similarly, there were both age- and sleep-dependent effects on limb retraction, as shown in Figure 1*E*, *F*. In Rest mice, hindlimb retraction was unchanged from ages 5 to 7 months (*t* = 0.6, not significant) yet worsened in the CSS group (*t* = 4.9, *p* < 0.001); and as observed with the ledge walk, CSS mice at 7 months evidenced poorer performances than Rest mice at 7 months (*t* = 3.9, *p* < 0.001). A second group of mice was used for locomotor, open field, and memory testing with *n* = 11 (8 male, 3 female/group). CSS-exposed mice showed increased locomotor activity for the first 2 min of the assay (*t* = 2.9 and *t* = 2.9, *p* < 0.05; Fig. 1*G*). CSS mice also showed increased relative time in the center:edges in the open field assay (*t* = 3.4, *p* < 0.01; Fig. 1*H*). With spatial memory testing also using an *n* = 11 as 8 male, 3 female/group, neither Rest nor CSS mice showed increased place preference for the recently moved object, relative to the unmoved object (*t* = 0.4 and *t* = 0.2, not significant, respectively; Fig. 1*I*). In contrast, only Rest mice showed place preference for a novel object (Rest, *t* = 6.7, *p* < 0.0001; CSS, *t* = 2.3, not significant; Fig. 1*J*), and CSS mice showed less preference for the novel object relative to Rest mice (*t* = 5.1, *p* < 0.0001). By 8 months, 2 of 11 CSS mice evidenced hunch spines, poor ambulation, and weight loss, whereas no mice within the Rest group evidenced severe motor deficits before 9 months of age. Overall, CSS accelerates deterioration in neurobehavioral performance in P301S mice, whereas short-term spatial memory is already impaired early in the course of disease.

### CSS results in a sustained increase in soluble tau, including oligomers

**Figure 2.**
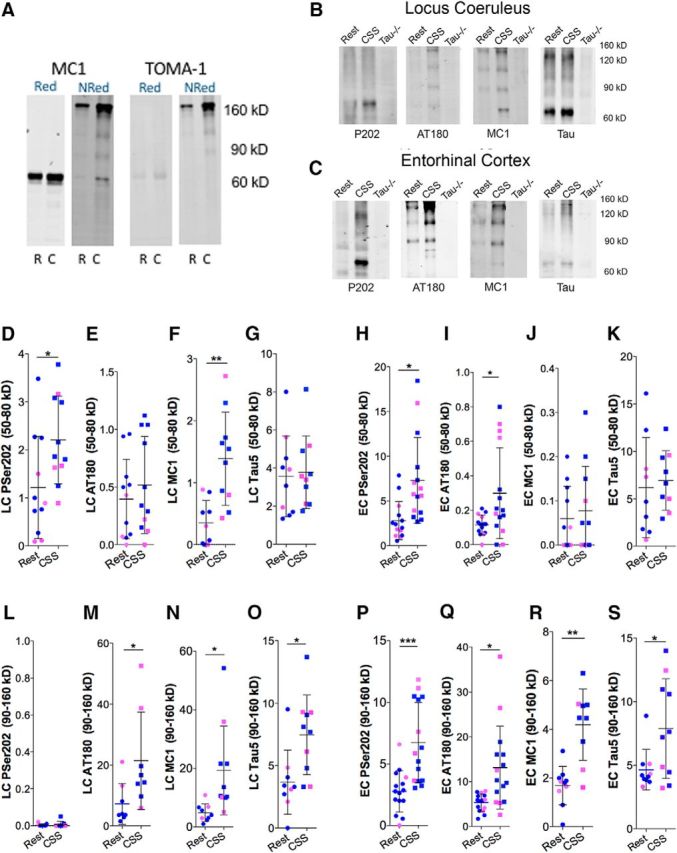
CSS results in lasting increases in soluble tau protein in the LC and EC. ***A***, Comparison of MC1 and oligomer (TOMA-1) tau immunoblots from same LC purified protein samples from a Rest P301S mouse (R) and a CSS P301S mouse (C) run under reducing (Red) conditions (left) and nonreducing (NRed) conditions (right). ***B***, Representative NRed LC gels (20 μg/lane) from Rest, CSS, and Tau^−/−^ mice for antibodies to phosphoserine 202 (P202), phosphothreonine 231 (AT180), MC1 tau (MC1), and total tau (Tau5). ***C***, Representative NRed gels from EC in Rest, CSS, and Tau^−/−^ mice for the same antibodies. ***D–G***, Individual normalized immunodensities at 50–80 kDa (monomeric) for LC lysates to P202, AT180, MC1, and Tau5. Individual data points: blue represents male; pink represents female. *n* = 9 (5 male, 4 female) for the LC samples. Error bars indicate mean ± SE. ***H–K***, Individual normalized immunodensities at 50–80 kDa for EC for the same antibodies and conditions where *n* = 14 (9 male, 5 female) for the EC samples. ***L–O***, Individual normalized immunodensities at 90–160 kDa for LC lysates to the same tau antibodies/conditions from the same gels analyzed for monomeric LC. ***P–S***, Individual normalized immunodensities at 90–160 kDa for EC lysates and antibodies, analyzed on gels used for monomeric analysis. Data were analyzed with unpaired *t* tests. *p < 0.05, **p < 0.01, ***p < 0.001.

The LC is one of the first sites with hyperphosphorylated tau, and the EC may be an early site of tau seeding (Braak et al., 2011; Kaufman et al., 2018). Soluble tau oligomers are implicated in both behavioral impairments and neurodegeneration (Santacruz et al., 2005). Nonreducing conditions have unveiled the presence of disulfide oligomeric tau, otherwise obscured in standard reducing gels (Fá et al., 2016). In preliminary studies, we compared reducing and nonreducing conditions for LC MC1 tau and found a robust shift to larger bands, 90–160 kDa under nonreducing conditions, bands that were undetectable in reducing conditions (Fig. 2*A*). The presence of oligomeric tau in nonreducing conditions was confirmed using an oligomer-specific tau antibody (clone TOMA-1), which also showed a prominent band near 160 kDa, without a signal in the reduced buffer (Fig. 2*A*). Thereafter, sustained effects of CSS on soluble tau in the LC and EC using nonreducing lysates were examined for monomeric (50–80 kDa) and oligomeric tau (90–160 kDa) in Rest and CSS mice, with *n* = 9 (5 male, 4 female)/group for LC and *n* = 14 (9 male, 5 female)/group for EC where protein was more abundant. For all tau antibodies assessed, lysates from Tau^−/−^ mice showed negligible immunoreactivity between 50 and 160 kDa. Representative Rest, CSS, and Tau^−/−^ images are provided in Figure 2*B*, *C*. P202 tau (50–80 kDa) was higher in mice exposed to CSS, relative to age-matched Rest mice in the LC (Fig. 2*D*; *t* = 2.3, *p* < 0.05) and in the EC, relative to Rest mice (Fig. 2*H*; *t* = 3.2, *p* < 0.05). In contrast, AT180 tau (50–80 kDa) was unchanged in the LC (Fig. 2*E*; *t* = 0.6, not significant) yet increased in EC (Fig. 2*I*; *t* = 2.6, *p* < 0.05). CSS increased monomeric LC MC1 tau (Fig. 2*F*; *t* = 3.8, *p* < 0.01) without affecting monomeric EC MC1 tau (Fig. 2*J*; *t* = 0.5, not significant). CSS did not influence monomeric tau5 in either the LC (Fig. 2*G*; *t* = 0.2, not significant) or EC (Fig. 2*K*; *t* = 0.4, not significant). We next examined the CSS response to tau oligomers specifically at 90–160 kDa bands. Oligomer bands were not detected for P202 in the LC (Fig. 2*B*,*L*). P202 oligomeric band density was evident in the EC and increased in response to CSS (Fig. 2*P*; *t* = 4.2, *p* < 0.001). CSS increased AT180 oligomers in the LC (Fig. 2*M*; *t* = 2.5, *p* < 0.05) and increased AT180 oligomers in the EC (Fig. 2*Q*; *t* = 2.8, *p* < 0.05). Similarly, CSS increased MC1 oligomers in both the LC (Fig. 2*N*; *t* = 2.3, *p* < 0.05) and the EC (Fig. 2*R*; *t* = 4.7, *p* < 0.001). Tau5 oligomers were also increased in both the LC (Fig. 2*O*; *t* = 2.8, *p* < 0.05) and the EC (Fig. 2*S*; *t* = 2.4, *p* < 0.05). Collectively, these findings demonstrate that CSS induces sustained increases in phosphorylated and MC1 tau, including soluble tau oligomers, in two regions with established heightened vulnerability in tauopathies, the LC and EC, and overall effect sizes on AT180, MC1, and Tau soluble oligomers are comparable for LC and EC.

### Sustained increase in tau pathology following CSS

**Figure 3.**
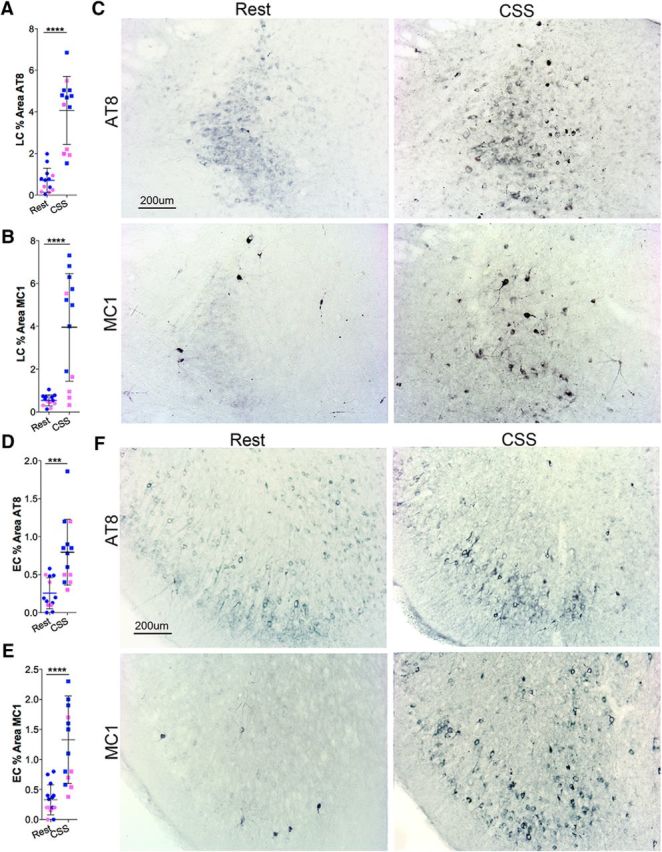
Lasting increases in AT8 and MC1 tau pathology within the LC and EC in response to CSS. ***A***, Individual percentage area with dense AT8 immunoreactivity within LC nucleus in Rest and CSS-exposed mice (*n* = 13: 8 male, 5 female/group). Individual data points: blue represents male; pink represents female. Black lines indicate mean ± SE. ***B***, Individual data points for percentage area with dense MC1-tau labeling within the LC nucleus for Rest and CSS-exposed mice (*n* = 13: 8 male, 5 female/group), labeled as in ***A***. ***C***, Representative images of AT8 (top) and MC1 (bottom) immunoreactivity labeled alkaline phosphatase (AP; navy blue) in 60 μm coronal sections from the LC from male mice exposed to Rest (left) and CSS conditions (right). ***D***, ***E***, Individual percentage area of AT8 (***D***) and MC1 (***E***) labeling in the EC in Rest and CSS-exposed mice (*n* = 8 male, 5 female/group). Data points are color-coded for sex as above. Error bars indicate mean ± SE. ***F***, Images in male mice for EC AT8 (top) and MC1 (bottom) panels in Rest (left) and CSS (right) mice. Data were analyzed with unpaired *t* tests. ***p < 0.001, ****p < 0.0001. Scale bar, 200 μm.

To determine whether CSS influences neuronal tau, we examined AT8 (PSer202 andPThr205 tau) and MC1 tau immunohistochemistry within the LC and several forebrain regions particularly sensitive to tau pathology: the EC, basolateral amygdala, and HC (Braak et al., 2011). To relate to soluble tau effects of CSS, mice were examined 6 months after the completion of CSS to identify lasting effects in *n* = 13 with 8 male and 5 female/group. P301S mice exposed to CSS had markedly greater AT8 within LCn than Rest P301S mice (Fig. 3*A*; *t* = 7.0, *p* < 0.0001). A similar response was observed for MC1 immunoreactivity in the LC in response to CSS (Fig. 3*B*; *t* = 4.9, *p* < 0.0001). Representative images in male mice are shown in Figure 3*C*. A similar response pattern was observed with increased AT8 in the EC, relative to Rest mice (*t* = 4.1, *p* < 0.001), and MC1 was increased in CSS-exposed P301S mice (*t* = 4.8, *p* < 0.0001). Thus, consistent with soluble tau findings, CSS results in sustained hyperphosphorylated tau and MC1 pathology in both the LC and EC.

**Figure 4.**
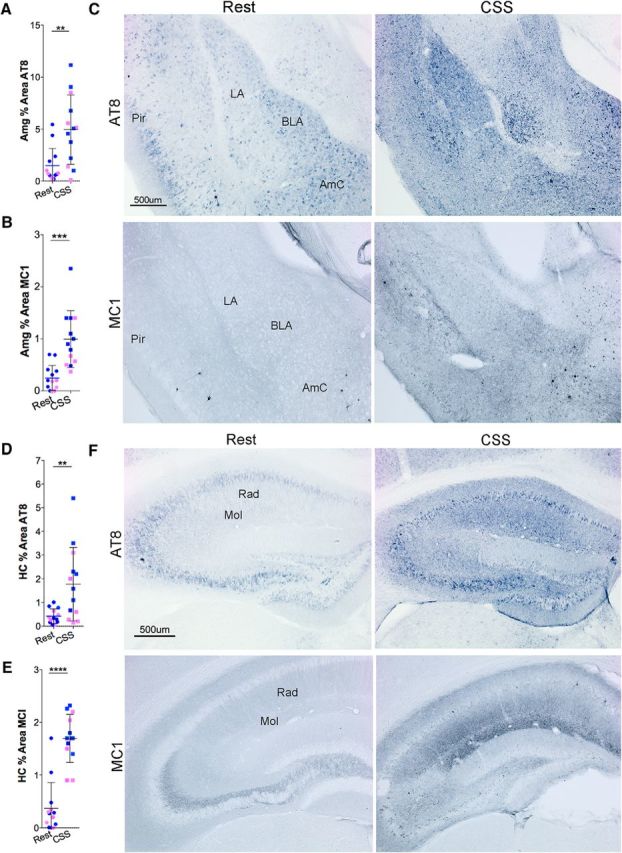
CSS results in sustained tau pathology in BLA/LA (Amg) and HC. ***A***, ***B***, Individual data points with *n* = 13 (8 male, 5 female) for amygdala percentage area with dense AT8 (***A***) and MC1 (***B***) labeling. Blue dots represent male; pink dots represent female. Black lines indicate group mean ± SE, analyzed with unpaired *t* tests. ***C***, Representative images of AT8 (top) and MC1 (bottom) IHC (blue represents AP) at bregma −1.70 in male Rest (left) and male CSS (right) mice. Regional references are as follows: LA and BLA nuclei within the amygdala, the amygdala cortex (AmC), and piriform cortex (Pir) in Rest mice. ***D***, ***E***, Individual data points for HC percentage area with dense AT8 (***D***) and MC1 (***E***) labeling. Blue dots represent male; pink dots represent female. ***F***, Six months after CSS exposure (*n* = 13: 8 male, 5 female). Black lines indicate group mean ± SE. ***C***, Representative images of AT8 (top, bregma −1.70) and MC1 (bottom, bregma −2.06) IHC (blue represents AP) in male Rest (left) and CSS (right) mice. Regional references are radiatum layer (Rad) and lacanosum moleculare (Mol) in the Rest mice. **p < 0.01, ***p < 0.001, ****p < 0.0001. Scale bars: ***C***, ***F***, 500 μm.

**Figure 5.**
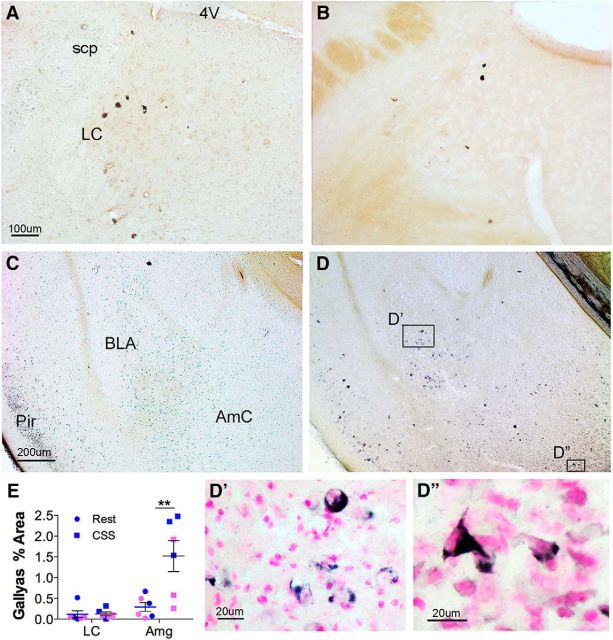
CSS upregulates Gallyas silver labeling. ***A***, ***B***, Low-power Gallyas silver impregnation signal in 60 μm coronal section of the LC in male Rest control (scp, superior cerebellar peduncle) (***A***) and CSS-exposed mice (***B***). ***C***, ***D***, Gallyas labeling within the piriform cortx (Pir), LA, and BLA cortex (AmC). ***D***, Section was then counterstained with nuclear fast red for nuclear visualization and reimaged at higher power (for insets ***D′***, ***D″***). ***E***, Individual data for percentage area of the LC and LA/BLA (Amg) with silver labeling. *n* = 6 (3 male, 3 female/group); blue represents male; pink represents female. Black lines indicate mean ± SE. Data were analyzed with one-way ANOVA and Bonferroni's *post hoc* analyses. **p < 0.01. Scale bars: ***A***, ***B***, 100 μm; ***C***, ***D***, 200 μm; ***D′***, ***D″***, 20 μm.

Findings were next extended to the BLA/LA and HC in the same groups of mice. CSS resulted in an increase in AT8 percent area within the amygdala (Fig. 4*A*; *t* = 3.4, *p* < 0.01) and in the HC (Fig. 4*D*; *t* = 3.1, *p* < 0.01). Examples of the tau immunohistochemistry are shown in Figure 4*C*, *F*. CSS also resulted in a sustained increase in MC1 in the amygdala (Fig. 4*B*; *t* = 4.5, *p* < 0.001) and the HC (Fig. 4*E*; *t* = 7.1, *p* < 0.0001). To gain insight into whether CSS influences the formation of NFTs, we next examined Gallyas silver staining within the LC and BLA/LA (amygdala) regions (*n* = 6: 3 male, 3 female/group). Despite the robust increase in AT8 and MC1 within LCn somata, only rare LCn were labeled with silver, and there was no effect of CSS on LCn Gallyas impregnation NFT-like inclusions (*t* = 0.0, not significant; Fig. 5*A*,*B*,*E*). In contrast, CSS significantly increased NFT-like inclusions in amygdala (*t* = 4.3, *p* < 0.01; Fig. 5*C–E*). Thus, CSS effects on AT8/MC1 tau immunoreactivity do not predict silver impregnation responses to CSS.

### CSS increases neuron loss within the LC and basolateral amygdala in the P301S murine model of tauopathy

**Figure 6.**
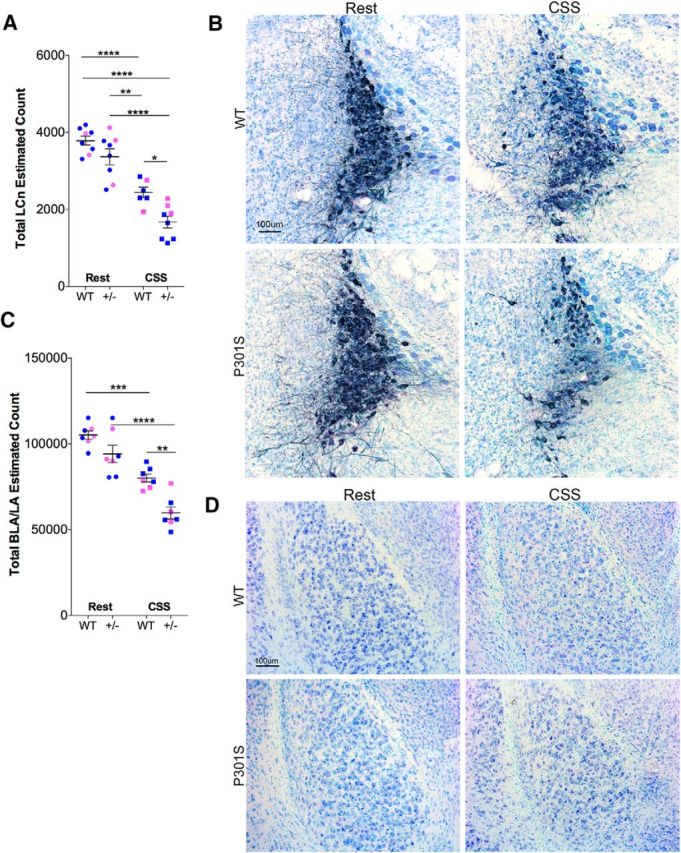
P301S mice show heightened susceptibility to CSS loss of LC and BL/LA neurons. ***A***, Individual LCn cell counts (*n* = 8: 5 male, 3 female/group) for unbiased optical fractionator estimates of total LCn in WT and P301S mice in response to Rest control conditions or CSS, followed by 6 months of recovery. Individual data: blue represents male; pink represents female. Black lines indicate mean ± SE. ***B***, Representative 60 μm coronal images of LC in male mice with TH^+^ immunoreactive neurons labeled (navy blue represents AP) and Giemsa stain (lighter blues) to highlight cells/nuclei. ***C***, Individual BLA/LA counts (blue represents male; pink represents female; *n* = 8: 5 male, 3 female/group) for stereological estimates. Black lines indicate mean ± SE. ***D***, Giemsa-labeled sections showing the BLA with boundaries delineated by external capsule fiber tracts. Data were analyzed with two-way ANOVA and Sidak's *post hoc* analyses. *p < 0.05, **p < 0.01, ***p < 0.001, ****p < 0.00001. Scale bars: ***B***, ***D***, 100 μm.

CSS induces degeneration in a subset of LCn in WT mice (Zhu et al., 2016). In light of the above observed effects of CSS on both soluble tau oligomers and tau pathology in the LC in P301S mice, we examined whether the P301S mutation increases susceptibility to CSS LCn degeneration by examining stereological counts in age-matched WT and P301S mutants exposed to CSS or Rest control conditions. Neurons within the confines of the LC nucleus were counted as TH^+^ or TH^−^ and analyzed collectively with *n* = 8 (5 male, 3 female/group). Consistent with previous reports, CSS induced loss of LCn in WT mice (Zhang et al., 2014; Zhu et al., 2016) (Tukey *q* = 8.0, *p* < 0.0001; Fig. 6*A*). Representative images are presented in Figure 6*B*. In Rest P301S mice, LCn counts were not significantly different from Rest WT (*q* = 2.9, not significant). A large reduction was observed, however, for P301S mice exposed to CSS (*q* = 11.0, *p* < 0.0001), so that LCn counts in P301S mice exposed to CSS were ∼30% lower than counts in CSS-exposed WT mice (*q* = 4.6, *p* < 0.05), demonstrating that CSS can further LCn degeneration in P301S mutant tau mice. Unable to obtain reliable (reproducible) counts of the densely packed EC neurons in layers II/III in 60 μm tissue sections, we examined the BLA/LA, where neurons were easy to isolate for counting in 60 μm sections, and boundaries for the BLA/LA are readily defined by surrounding white matter tracks and distinct neuronal morphologies (Chareyron et al., 2011). Sample sizes were *n* = 7 (4 male, 3 female/group). There was no genotype effect under Rest conditions on BLA/LA neurons (*q* = 3.2, not significant; Fig. 6*C*). Representative images of the BLA/LA are shown in Figure 6*D*. CSS resulted in a significant reduction in BLA/LA in WT mice (*q* = 7.2, *p* < 0.001) and in P301S mice (*q* = 9.9, *p* < 0.0001), so that the BLA/LA neuron estimates in P301S mice following CSS were significantly lower than in WT mice after CSS (*q* = 5.9, *p* < 0.01). Overall, neuron loss in both the LC and BLA/LA occurs in response to CSS, with both LCn counts and BLA/LA counts lower in P301S mice exposed to CSS than in WT mice exposed to CSS.

### CFS also hastens neurobehavioral decline and tau pathologic changes

**Figure 7.**
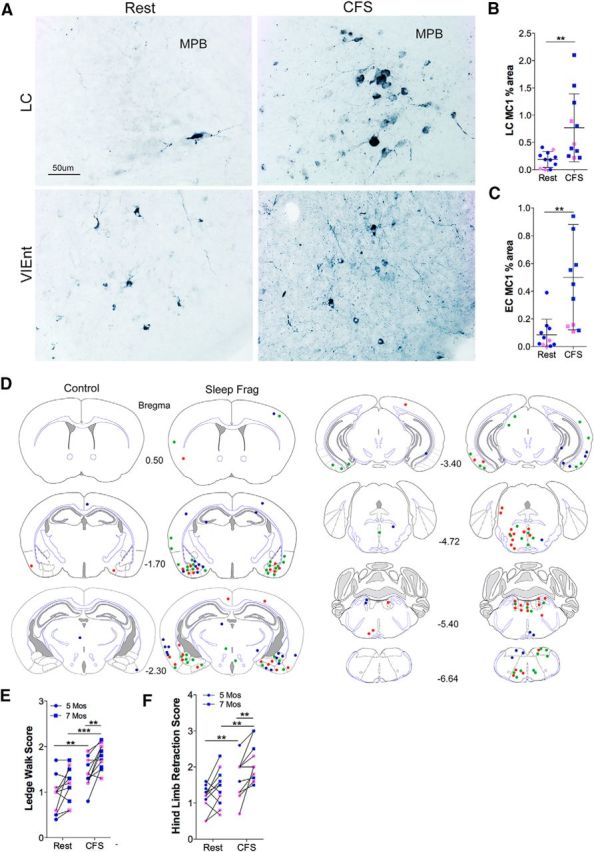
CFS increases MC1 tau pathology in susceptible regions and worsens motor performance. ***A***, Representative images of MC1 labeling in LC (top) and ventrolateral EC (VlEnt, bottom). Medial parabrachialis nucleus (MPB) is highlighted to the right of the LC nucleus. ***B***, ***C***, Individual MC1 percent area (blue represents male; pink represents female) for LC nucleus (***B***) and EC (***C***) with dense immunoreactivity (blue represents AP; navy represents Rest; *n* = 11: 8 male and 3 female/group). ***D***, Composite brain mapping of MC1 tau densely labeled neurons in 3 mice 6 months after conditions of Rest and CFS, highlighting the three individual responses as red, green, or blue dots marking labeling sites. ***E***, ***F***, Group scores (Rest; *n* = 11: 8 male, 3 female) for ledge walk (***E***) and hindlimb retraction for ages 5 months (closed circles) and 7 months (closed squares). Data were analyzed with two-way ANOVA and Sidak's *post hoc* analyses. *p < 0.05, **p < 0.01, ***p < 0.001. Scale bar, 50 μm.

Because the CSS paradigm increases spontaneous exploratory behavior in a novel environment, which could confound effects of sleep disruption, we also explored the effects of CFS on MC1 tau immunohistochemistry and motor performance in *n* = 11 mice (8 male, 3 female/group). Overall, CFS increased LC and EC MC1, as illustrated in Figure 7*A–C*. Relative to Rest mice, MC1 percent area increased in the LC (*t* = 3.0, *p* < 0.01; Fig. 7*B*) and also in the EC (*n* = 11: 8 male, 3 female/group; Fig. 7*C*; *t* = 3.5, *p* < 0.05). We then mapped densely labeled MC1 somata across the brain and found that the density of MC1-labeled neurons in CFS exposed mice was greater overall within areas also affected in Rest mice, as summarized in the brain maps of MC1 neurons (Fig. 7*D*). With the ledge walk test, there was no progression observed in the Rest (*t* = 2.4, not significant), whereas CFS-exposed mice from 5 to 7 months deteriorated in performance (*t* = 3.8, *p* < 0.01). There were also differences in ledge walk scores between the Rest and CFS mice, at 5 months (*t* = 3.3, *p* < 0.01) and at 7 months (*t* = 4.2, *p* < 0.001). Similarly, there were CFS effects on hindlimb retractions scores for the mice at 5 months and 7 months (*t* = 2.7, *p* < 0.05 and *t* = 3.6, *p* < 0.01, respectively) with no progression in mice for Rest (*t* = 2.2, not significant), yet a progression in CSFS-exposed mice CSS (*t* = 3.8, *p* < 0.01).

### Sleep disruption activates glia within regions of tau pathology in P301S mice

**Figure 8.**
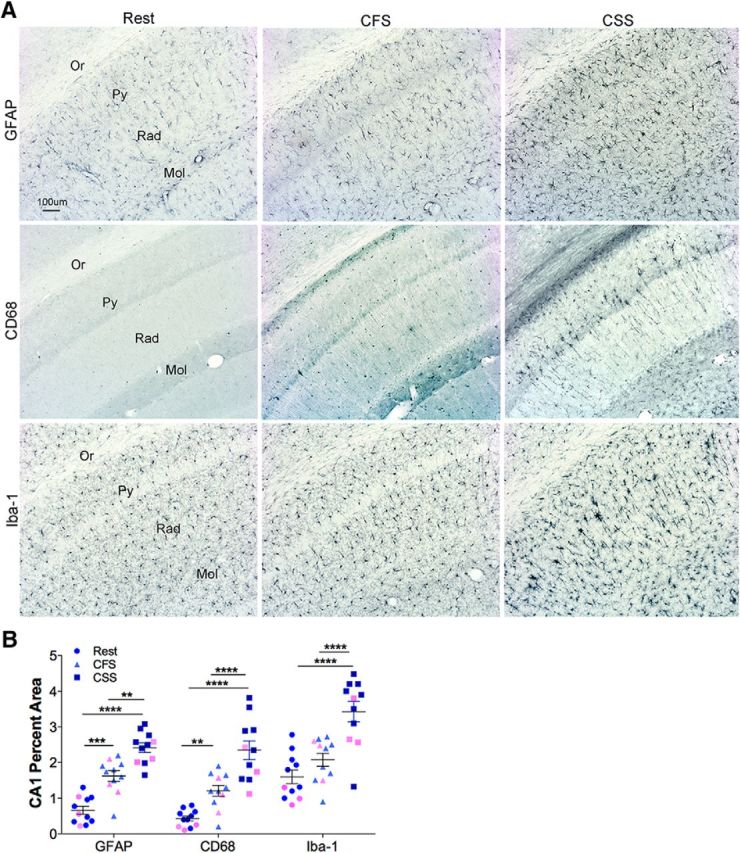
Persistent glial activation in response to chronic sleep disruption in both CSS and CFS. ***A***, Representative images of GFAP, CD68, and Iba-1 immunoreactivity (AP blue) in HC lateral CA1 at bregma −2.18, across conditions in male mice, Rest (left panels), CFS (middle panels), and CSS (right panels). ***B***, Percentage area coverage data are presented. Individual responses: blue represents male; pink represents female. Error bars indicate mean ± SEM. *n* = 11 (8 male, 3 female) for each sleep condition. Data were analyzed with two-way ANOVA and Sidak's *post hoc* analyses. **p < 0.01, ***p < 0.001, ****p < 0.00001. Scale bar: ***A***, 100 μm.

Astrocytes and/or microglia are implicated in synapse loss, tau propagation, and neurodegeneration in tauopathies (Asai et al., 2015; Hong et al., 2016; Liddelow et al., 2017). As indices of microglial and astrocyte activation, we examined the percentage area coverage for astrocyte-specific (GFAP) and microglial-specific (Iba-1) and CD68 immunoreactivity within the HC, as a representative tau-susceptible region. Having identified increased tau pathology in the HC in both CSS and CSF mice, we examined both forms of sleep disruption here, matching sexes across sleep condition with *n* = 11 (8 male, 3 female/group). GFAP percentage coverage of CA1 was increased in CFS, relative to Rest (*q* = 5.3, *p* < 0.001) and in CSS relative to Rest (*q* = 9.6, *p* < 0.0001). GFAP signal was higher in CSS than in CFS (*q* = 4.3, *p* < 0.01, as summarized in Fig. 8*B*). Overall results were similar for CD68, where CD68 increased in CFS relative to Rest (*q* = 4.3, *p* < 0.01) and was further increased in CSS relative to Rest (*q* = 10.5, *p* < 0.0001), so that CD68 in CSS was higher than in CFS (*q* = 6.2, *p* < 0.0001). There was no significant increase in Iba-1 for CFS relative to Rest (*q* = 2.6, not significant), although there was a large increase in Iba-1 percent area in CSS mice, relative to Rest (*q* = 10.0, *p* < 0.0001) and relative to CFS (*q* = 7.4, *p* < 0.0001). Collectively, the data show that both astrocyte and microglial reactivity is evident in a sustained fashion following early life sleep disruption.

### CSS increases MC1 tau in the LC acutely, whereas effects of CSS on cortical tau are evident only over time

**Figure 9.**
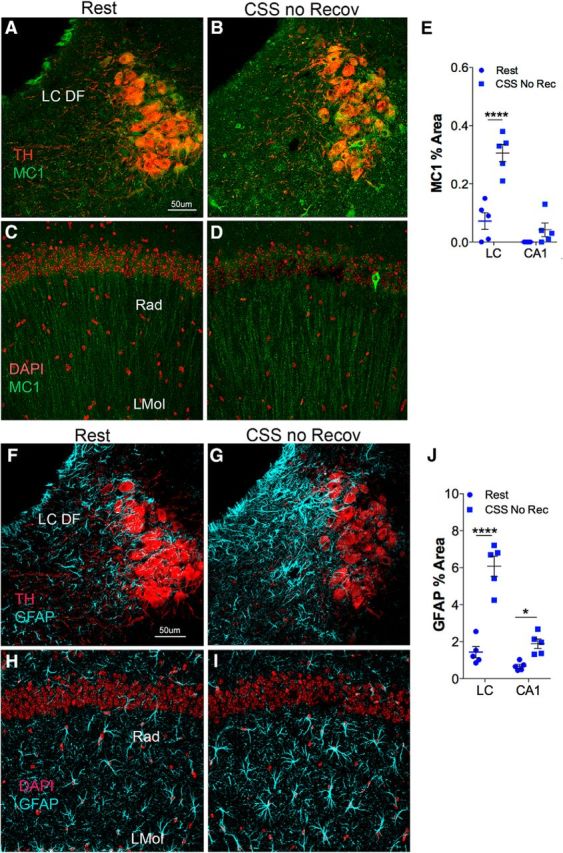
Immediate response to CSS in P301S mice varies for LC and HC. ***A***, ***B***, Confocal images of MC1 (green) with TH-labeled (red) LCn in Rest-exposed (***A***) and CSS-exposed (***B***) mice immediately after exposures. Regional reference, LC dendritic field (LC DF). ***C***, ***D***, Confocal images of MC1(green) in CA1 HC with DAPI labeling (red) of nuclei to highlight pyramidal neurons. Reference areas are radiatum layer (Rad) and lacanosum moleculare (Mol). ***E***, Individual MC1 percent area (with black lines indicating mean ± SE) in the LC and CA1 region of HC. ***F***, ***G***, Confocal images of GFAP (blue) with TH-labeled (red) LCn from the same sections (tripled labeled) shown in ***A*** and ***B***, revealing a striking GFAP increase over LC DF in response to CSS. ***H***, ***I***, Confocal images of GFAP (blue) in CA1 HC with DAPI labeling (red) of nuclei from the same sections imaged for MC1 and DAPI in ***C*** and ***D***. ***J***, Individual GFAP percentage area (with mean ± SE) in LC and CA1. *p < 0.05, ****p < 0.0001. Scale bars: ***A–D***, ***F–I***, 50 μm.

A second set of mice was randomized to CSS or Rest conditions for 4 weeks and then examined for tau and glial responses immediately following CSS within the LC and HC (*n* = 5 males/group). Representative images of MC1 in the two regions are presented in Figure 9*A–D*. CSS increased MC1 coverage within the LC (*q* = 9.8, *p* < 0.0001) without increasing MC1 immunoreactive area in the HC (*q* = 1.8, not significant), as summarized in Figure 9*E*. Within the same sections, examined as a triple label, CSS resulted in an immediate increase in GFAP coverage within the LC (*q* = 9.6, *p* < 0.0001) and increased GFAP coverage in the HC (*q* = 2.5, *p* < 0.05; Fig. 9*J*). Representative images of the GFAP response in the same sections imaged for the MC1 response (Fig. 9*A–D*) are presented in Figure 9*F–I*. In summary, P301S mice evidence an early MC1 tau pathology response in the LC, whereas CSS effects in the HC develop only after time, and astrocyte reactivity early on appears more pronounced in the LC than in the HC.

## Discussion

Sleep loss increases brain amyloid-β (Aβ) levels and Aβ amyloid plaque in transgenic mouse models of AD (Kang et al., 2009; Xie et al., 2013). These sleep loss effects on Aβ and amyloid are believed to be exclusively extracellular and limited to the forebrain. In AD, however, tau is implicated in amyloid-induced neural injury (Roberson et al., 2007; Ittner et al., 2010). Yet effects of sleep loss on tau and the progression of tauopathy have been largely unexplored. The present studies examined the effects of chronic sleep disruption on tau protein biochemistry, neuroanatomy, and behavior in a murine model overexpressing human P301S mutant tau. We found that early life CSS advanced the temporal progression of tauopathy, manifesting as a worsening of neurobehavioral impairment and sustained increases in soluble tau oligomers, AT8 and MC1 tau pathology within the LC, HC, EC, and other regions susceptible to tau accumulation, and greater NFT in the amygdala. Moreover, CSS furthered neurodegeneration of LC and amygdala neurons and activated glia in tau-affected regions, with all of these effects evident months after CSS. A second form of sleep disruption, CFS, also advanced neurobehavioral impairment and increased tau pathology. Collectively, the findings identify chronic early life sleep disruption as an important modifier of P301S tauopathy and demonstrate that chronic sleep disruption also has important effects on intraneuronal tau protein processing, in addition to the previously described sleep loss effects on extracellular Aβ and amyloid.

How would early life sleep disruption induce sustained advancement of tauopathy? We propose several possible mechanisms that may act independently or synergistically to advance the progression of tauopathy. Both paradigms of sleep disruption can result in loss of sirtuin Type 1 (SirT1) in select neurons, including LCn (Zhu et al., 2015, 2016). Deficiency of SirT1 was recently shown to worsen neurobehavioral impairments in P301S mice (Min et al., 2018). Thus, a lasting reduction in SirT1 may contribute to the persistent pathologic tau in the present study. Additionally, CSS induced immediate and sustained misfolding of tau, as evidenced by increased MC1 tau pathology within the LC and increased soluble phosphorylated and misfolded (MC1) tau in the EC. The LC is implicated as an early site for misfolded tau in tauopathy and a site from which pathologic tau can propagate (Braak et al., 2011; Iba et al., 2015). There is, however, a recent report suggesting that the EC, but not the LC, is involved early in tau seeding (Kaufman et al., 2018). As CSS increased EC tau in the present study, it is also possible that sleep loss increases tau seeding directly from the EC. In addition, CSS induced significant degeneration of LCn, particularly in P301S mutants, and lesioning of the LC can have lasting effects on the progression of tauopathy. Specifically, early life lesioning of LC neurons (at the same age of our CSS exposure) in P301S mice results in more profound neurobehavioral deficits, in particular memory, and increased gliosis, without increasing tauopathy (Chalermpalanupap et al., 2018). Consistent with this finding, we observed increased impairment in memory and a striking gliosis in response to CSS. Interestingly, LC lesioning does not appear to influence tau pathology, whereas early life sleep disruption does. This may have to do with differences in antibodies used to assess tau pathology, where the only tau species examined after LC lesioning was phosphorylated tau at Ser 306 and 404 (Chalermpalanupap et al., 2018). Alternatively, the composite findings we observe with CSS may include LCn injury and may also include the metabolic resetting as mentioned above. Finally, we note a persistent microglial activation in tau-affected regions. Microglial activation in murine tauopathy can worsen tau spreading and pathology (Maphis et al., 2015). In summary, any one or more of these possibilities may contribute to the persistent progression of tauopathy after CSS and CFS.

CSS increased levels of specific post-translational modifications of tau in P301S mice, which are known to influence the severity of tauopathy in murine models and in humans. Specifically, CSS increased tau phosphorylated at threonine 231 (P231) and MC1 tau in both the LC and EC. In AD, increased P231 tau levels in the CSF predict lower hippocampal volumes and greater declines in volume over time (Hampel et al., 2005), and P231 tau levels predict cognitive decline in individuals with mild cognitive impairment (Buerger et al., 2002). P231 tau disrupts tubulin intermolecular binding in axons, which may contribute to neuronal injury and demise (Moszczynski et al., 2015; Schwalbe et al., 2015). Additionally, P231 tau is critical for the formation of tau fibrils (Moszczynski et al., 2015). Increases in MC1 tau were also evident following CSS. MC1 antibody detects a pathological conformational change in tau protein, one that is identified early on in AD and is not detected in normal brains (Weaver et al., 2000). Importantly, its presence also positively correlates with the severity and progression of AD (Mead et al., 2016). Remarkably, CSS resulted in sustained increases in pathogenic tau oligomers, evident at least 6 months after cessation of CSS. This suggests that early life CSS imparts a lasting metabolic resetting in affected neurons, in at least the two regions assayed: the LC and EC. That the same lysate run under nonreducing conditions evidenced oligomeric bands and when processed under reducing conditions revealed a monomeric band supports reversible oxidative tau oligomerization. Disulfide cross-linked tau dimers can aggregate and propagate and are implicated in tauopathy progression (Kim et al., 2015). Thus, CSS results in important tau modifications that are implicated in cognitive decline and neural injury in AD, where one of the CSS-induced tau modifications is reversible oxidative oligomerization.

P301S mice showed heightened vulnerability to CSS-induced neurodegeneration in both regions examined: the LC and BLA/LA. In the LC in P301S mice, CSS neuron loss does not appear to require NFT as the LC in CSS-exposed mice exhibited rare tangles. LCn loss was associated with an increase in LC-soluble tau oligomers and glial activation. Our findings are in keeping with the previously described temporal progression of tauopathy in P301S mice, where neurofibrillary formations occur well after synaptic loss and gliosis (Yoshiyama et al., 2007). There is recent *in vivo* evidence that oligomers, rather than NFT, parallel neuron loss in tauopathy. Specifically, reducing RNA binding protein TIA1 in P301S mice reduces soluble oligomers and in parallel limits neuronal loss, yet this intervention increased NFT (Apicco et al., 2018). A next important step will be to test the role of disulfide oligomerization in CSS-induced neuron loss in both WT and P301S mice.

Both CSS and CFS increased the severity of motor deficits, the density of tau pathology, and the intensity of both astrocyte and microglial activation, supporting the concept that chronic disruption of sleep, rather than repeated exposure to a novel environment and/or increased ambulation as in CSS, advances tauopathy in the P301S mice. We cannot compare magnitudes of responses, as there is no way to equate severity of sleep disruption across the two models. There were, however, relative differences in the overall response patterns that may provide insight into how the type of sleep disruption may influence the phenotype of tauopathy. Interestingly, the motor deficits started sooner following CFS, whereas EC tau pathology and glial activation appeared more pronounced in response to CSS. We propose that, although chronic sleep disruption (both CSS and CFS) can advance tauopathy, there may be unique metabolic responses to the different forms of sleep disruption (with different patterns of wake durations) which influence the behavioral and pathology outcomes in tauopathy, and these unique responses may contribute to heterogeneity of pathology and neurobehavioral signs across individuals with a given tauopathy. Alternatively, differences in severity of sleep disruption may influence response patterns.

Both CFS and CSS resulted in sustained glial activation. CSS increased microglial activation as evidenced by increased CD68 and Iba-1 percent area, whereas CFS increased only CD68 and not Iba-1. While both Iba-1 and CD68 increases are used to characterize microglial activation, in AD, Iba-1 response can be less robust than the CD68 response (Hendrickx et al., 2017). Thus, both forms of sleep disruption activate microglia. Microglial activation is an early sign in tauopathy, including in the P301S mice, where microglial activation precedes tangle formation (Yoshiyama et al., 2007), but this response may also contribute to disease. Specifically, microglia can propagate tau, whereas depletion of microglia can markedly reduce propagation of tau pathology in a mouse model (Asai et al., 2015). Astrocyte activation was more prominent in CSS than in CFS, at least in the HC, where compared. It is of interest that brain regions with increased MC1 deposition were also regions with increased astrocyte activation, suggesting that these two responses are tightly linked across Rest and CSS conditions.

Collectively, the present findings demonstrate that chronic sleep disruption in early adult life advances the temporal progression of tauopathy. Insufficient sleep in adolescents is increasingly common in modern societies (Keyes et al., 2015). CFS is observed in all cases of obstructive sleep apnea, a disorder with increasing prevalence in adolescents and young adults (Peppard et al., 2013; Güngör, 2014). The above findings suggest that these common forms of chronic sleep disruption may prove to be important and modifiable factors in the progression of tauopathies, including AD.
